# Diverse Effects of Lead Nitrate on the Proliferation, Differentiation, and Gene Expression of Stem Cells Isolated from a Dental Origin

**DOI:** 10.1155/2014/235941

**Published:** 2014-01-27

**Authors:** Mariam Abdullah, Fazliny Abd. Rahman, Nareshwaran Gnanasegaran, Vijayendran Govindasamy, Noor Hayaty Abu Kasim, Sabri Musa

**Affiliations:** ^1^Department of Conservative Dentistry, Faculty of Dentistry, University of Malaya, 50603 Kuala Lumpur, Malaysia; ^2^Hygieia Innovation Sdn. Bhd, Lot 1G-2G, Komplex Lanai, No. 2, Persiaran Seri Perdana, Persint 10, 62250 Putrajaya, Malaysia; ^3^Department of Children's Dentistry and Orthodontics, Faculty of Dentistry, University of Malaya, 50603 Kuala Lumpur, Malaysia

## Abstract

Lead (Pb^2+^) exposure continues to be a significant public health problem. Therefore, it is vital to have a continuous epidemiological dataset for a better understanding of Pb^2+^ toxicity. In the present study, we have exposed stem cells isolated from deciduous and permanent teeth, periodontal ligament, and bone marrow to five different types of Pb^2+^ concentrations (160, 80, 40, 20, and 10 *µ*M) for 24 hours to identify the adverse effects of Pb^2+^ on the proliferation, differentiation, and gene expression on these cell lines. We found that Pb^2+^ treatment altered the morphology and adhesion of the cells in a dose-dependent manner. There were no significant changes in terms of cell surface phenotypes. Cells exposed to Pb^2+^ continued to differentiate into chondrogenesis and adipogenesis, and a severe downregulation was observed in osteogenesis. Gene expression studies revealed a constant expression of key markers associated with stemness (Oct 4, Rex 1) and DNA repair enzyme markers, but downregulation occurred with some ectoderm and endoderm markers, demonstrating an irregular and untimely differentiation trail. Our study revealed for the first time that Pb^2+^ exposure not only affects the phenotypic characteristics but also induces significant alteration in the differentiation and gene expression in the cells.

## 1. Introduction

Lead (Pb^2+^) is regarded as one of the most toxic substances among heavy metals. It is derived from the environment as well as an industrial pollutant. Pb^2+^ causes serious illness which covers not only physiological and biochemical dysfunctions but also behavioural dysfunctions in humans [1, 2]. The impact is more prevalent in children [3] who have a high tendency to accumulate Pb^2+^ in their circumpulpal dentin [4]. The common route of exposure of Pb^2+^ is in the blood and eventually it is deposited in the hard tissues such as bone and teeth. The deposition in the latter tissue is permanent [5]. Therefore, teeth can be a useful long-term record of Pb^2+^ accumulation [6] and have been used as biological markers to environmental pollution [7–15].

Several factors are identified which influence the Pb^2+^ deposition in teeth. These include types of teeth and the presence of caries. The deposited Pb^2+^ in teeth during mineralization is largely retained since the teeth have hard stable tissues [16]. Pb^2+^ is distributed with the highest concentration in the circumpulpal dentin which is located in the innermost layer of dentin, adjacent to the dental pulp. The mechanism by which Pb^2+^ is deposited within the matrix of the primary and circumpulpal dentin is not clear although both primary and circumpulpal dentins accumulate Pb^2+^ with increased exposure and with postnatal age [17]. Nevertheless, it has been suggested that the deposition of Pb^2+^ in teeth is probably due to the similar oxidation number of both Ca^2+^ and Pb^2+^ ions. Studies also have shown that the toxicity effect of Pb^2+^ becomes apparent due to the ability of the metabolic cation Pb^2+^ to bind with specific ligands of biomolecular substances that play a vital role in various physiological functions [18, 19]. It also competes with ion transport and interferes with normal physiology [20]. There are also studies on the effect of Pb^2+^ on cells derived from human periodontal ligament [21]. This is due to availability of material and because of the incorporation of elements into the mineral phase of dental tissues [22].

Stem cells play an important role in maintaining the homeostasis and function of tissues and play a pivotal role in dealing with invaders such as Pb^2+^. This effect has been documented in previous work that described the effects of Pb^2+^ on dental cells [23].

We have studied the effects of various types of stem cells from a dental origin, namely, from deciduous teeth (SCDs), permanent teeth (DPSCs), and periodontal ligament (PDLs) as well as from bone marrow (BM-MSCs) against various Pb^2+^ concentrations with respect to their proliferation, multilineage differentiation capacity, and gene expression level. Interestingly, though all stem cells are almost similar to each other, we discovered significant differences in terms of proliferation and differentiation of stem cells derived from SCD, DP, PDL, and BM.

## 2. Materials and Methods

### 2.1. Tissue Collection and Isolation of Cells

This work was approved by the Medical Ethics Committee, Faculty of Dentistry, University of Malaya (medical ethics clearance number: DF CO1107/0066 (L)). All the subjects consented to the study. All subjects were nonsmokers, nonalcoholics, and free from any infectious diseases such as HIV, HBV, and HCV.

Bone Marrow (BM) stem cells or mesenchymal stem cells (MSCs) cultures were established from three donors (age: 24–35) as previously described [24]. Briefly, 60 mL BM was aspirated aseptically from the iliac crest of each patient under deep sedation. All processings of samples were done inside a class 100 biosafety hood. The BM was diluted (1 : 1) with knockout Dulbecco's modified Eagle's medium (KO-DMEM; Invitrogen, Carlsbad, CA, USA; http://www.invitrogen.com/) and centrifuged at 1800 rpm for 10 minutes to remove anticoagulants and other debris. The supernatant was discarded and the BM washed once with the culture medium. Mononuclear cells (MNCs) were isolated by layering onto a lymphoprep density gradient (1 : 2; Axis-Shield PoCAS). The MNCs present in the buffy coat were washed again with the culture medium. The mononuclear fractions that also contained MSCs were plated into culture flasks.

Dental pulp stem cells (DPSCs) cultures were established from sound intact third molars from adults (age: 24–35) and deciduous (SCDs) were established from molars from children (age: 3–12) as previously described [25]. Periodontal ligament (PDLs) tissues were separated from the root surface of deciduous teeth using a sterilized scalpel. Briefly, root surfaces were cleaned with Povidone-iodine (Sigma Aldrich, St. Louis, MO, USA; http://www.sigmaaldrich.com) and the pulp was extirpated within two hours of postextraction and then processed as follows. The pulp tissues as well as PDL tissues were minced into small fragments prior to digestion in a solution of three mg/mL collagenase type I (Gibco, Grand Island, NY, http://www.invitrogen.com) for 40 minutes at 37°C. After neutralization with a 10% Fetal Bovine Serum ( FBS, Fetal Bovine Serum, (FBS) (Hyclone; Thermo Scientific Inc, Waltham, MA; http://www.thermoscientific.com), the cells were centrifuged and were seeded in a culture flask.

All cells were cultured in an identical culture condition videlicet, in T75 cm^2^ culture flasks (BD Pharmingen, San Diego CA, USA; http://www.bdbiosciences.com) with a culture medium containing KO-DMEM, 0.5% and 10000 *μ*g/mL of penicillin/streptomycin (Invitrogen), 1% 1x Glutamax (Invitrogen) and 10% FBS with a humidified atmosphere of 95% of air, and 5% of CO_2_ at 37°C. Nonadherent cells were removed 48 h after initial plating. The medium was replaced every three days until the cells reached 80–90% confluency.

### 2.2. Preparation of Pb^2+^

The stock concentration of Pb^2+^ was prepared by dissolving Pb^2+^ nitrate (Pb [NO_3_]_2_) (Cat No 203580, Sigma Aldrich) in distilled water at a concentration of 10^-4 ^M. A total of 5 different Pb^2+^ concentrations (160, 80, 40, 20, and 10 *μ*M) were used in this study with 50% ratio between each concentration. The concentration of Pb^2+^ was designed based on previous study [26].

### 2.3. Exposure of Cell Lines to Pb^2+^

The BM-MSCs, SCDs, DPSCs, and PDLs were seeded at 1000 cells/cm^2^ in the 6-well plates (BD Bioscience) and allowed to grow for 96 hours. Then freshly prepared Pb^2+^ solution was added into the culture media to obtain the aforesaid concentration. Exposed cells were cultured for 24 hours before they were used for the subsequent experiments.

### 2.4. Growth Kinetics

Exposed cell lines with Pb^2+^ were counted and assessed for viability using a trypan blue dye exclusion technique. The population doubling time (PDT) of Pb^2+^ exposed BM-MSCs, PDLs, SCDs, and DPSCs were analyzed using the formula:

(1) $$\begin{array}{c} \text{PDT}=\frac{t\text{plg2}}{\left(\text{lgNH}-\text{lgNI}\right)}, \end{array}$$

where NI is the inoculum cell number, NH is the cell harvest number, and *t* is the time of the culture (in hours).

### 2.5. MTT Assay

MTT assay was performed as described previously [27]. Each experiment was done in triplicate. The relative cell viability (%) related to control wells containing cell culture medium without nanoparticles or PBS as a vehicle was calculated by [*A*]test/[*A*]control × 100, where [*A*]test is the absorbance of the test sample and [*A*]control is the absorbance of control sample.

### 2.6. LDH Assay

LDH assay was performed as described previously [27]. Each experiment was done in triplicate. The spectrophotometer was calibrated to zero absorbance using culture medium without cells. The relative LDH leakage (%) related to control wells containing cell culture medium without nanoparticles or PBS as a vehicle was calculated by [*A*]test/[*A*]control × 100, where [*A*]test is the absorbance of the test sample and [*A*]control is the absorbance of the control sample.

### 2.7. Senescence Associated with *β*-Galactosidase Assay

The Pb^2+^ exposed BM-MSCs, PDLs, SCDs, and DPSCs were tested for senescence-associated *β*-galactosidase (SA-*β*gal) staining with the SA-*β*gal staining kit (Sigma Aldrich) and used according to the manufacture's instructions. Briefly, all cell lines were washed twice with DPBS (–Ca^2+^, –Mg^2+^, Invitrogen) and incubated with 1x fixative solution for 15 minutes at room temperature. Subsequently, the fixed cells were rewashed using 2 mL of DPBS (–Ca^2+^, –Mg^2+^, Invitrogen) and stained with 1 mL of the staining solution mixture overnight at 37°C in a dry incubator. The development of the blue colour was observed under a phase contrast microscope and the quantitative analyses of the SA-*β*gal staining were done by counting the percentage of blue-stained cells that represent senescence cells in the selected field of each sample.

### 2.8. Immunophenotyping Analysis

Pb^2+^ exposed cell lines were subjected to immunophenotyping analysis by using a flow cytometry. A total volume of 200 *μ*L of a cell suspension (1 × 10^5^ cells) was incubated with the labeled antibodies in the dark for 1 hour at 37°C. The following antibodies were used to mark the cell surface: epitopes-CD90-phycoerythrin (PE), CD44-PE, CD73-PE, CD166-PE and CD34-PE, and CD45-Fluoro-isothyocyanate (FITC), and HLA-DR-FITC (all from BD Pharmingen). All the analyses were standardized against a negative control of cells incubated with isotype-specific IgG1-PE and IgG1-FITC (BD Pharmingen). At least 10,000 events were acquired on Guava Technologies flow cytometer and the results were analyzed using Cytosoft, Version 5.2, from Guava Technologies.

### 2.9. Differentiation Assay

The Pb^2+^ exposed cell lines were replated at a density of 1000 cells/cm^2^ in 6-well plates and were grown to confluence and subjected to differentiation into adipogenic, chondrogenic, and osteogenic lineages according to the method described earlier [24]. The adipogenic lineage was initiated in a 3-week culture period by inducing the cells with 10% FBS, 200 *μ*M indomethacin, 0.5 mM 3-Isobutyl-1-methylxanthine (IMBX), 10 *μ*g/mL insulin, and 1 *μ*M dexamethasone (all reagents from Sigma Aldrich). Lipid droplets were visualized by staining with Oil Red O staining (Sigma Aldrich). For the chondrogenesis differentiation, the cells were cultured in a media supplemented with ITS+1 (Sigma Aldrich), 50 *μ*M of L-ascorbic acid-2 phosphates, 55 *μ*M of sodium pyruvate (Invitrogen), 25 *μ*M of L-proline (Sigma Aldrich), and 10 ng/mL of the transformation growth factor-beta (TGF-*β*) (Sigma Aldrich). Assessment of the proteoglycans accumulation was visualized by the Alizarin Blue staining (Sigma Aldrich). The osteogenic differentiation was stimulated in a 3-week culture period in a media supplemented with 10% FBS, 10^−7^ M dexamethasone, 10 mM-glycerol phosphate (Fluka, Buchs, Switzerland), and 100 *μ*M of L-ascorbic acid-2 phosphate. The assessment of calcium accumulation was visualized by using the von Kossa staining technique (Sigma Aldrich).

### 2.10. Reverse Transcription Polymerase Chain Reaction (RT-PCR)

Total RNA from Pb^2+^ exposed BM-MSCs, PDLs, SCDs, and DPSCs was isolated with Trizol (Invitrogen) and used according to the manufacturer's instructions. The RNA of the Pb^2+^ exposed cells was converted to cDNA with Superscript II to reverse transcriptase (Invitrogen) and used according to the manufacturer's instructions. cDNA amplification was performed in a thermocycler at 94°C/1 min, a 58°C/30 sec, and 72°C/1 min. The expressions of ectoderm, endoderm, and pluripotent genes were quantified in duplicate with SYBR green master mix (Applied Biosystem, Foster City, CA, USA). For data analysis, the comparative CT method was used. For RT-PCR, the products were resolved on 1.5% agarose (Invitrogen) gel and was run in 1x Tris borate-EDTA buffer. The primer sequences are tabulated in Supplementary Table 1 (see the Supplementary Materials available online at http://dx.doi.org/10.1155/2014/235941).

### 2.11. Soft Agar Colony Formation Assay

BM-MSCs, PDLs, SCDs, and DPSCs cells exposed to Pb^2+^ were tested for soft agar assay colony formation assay (Cells Biolab, INC) and used according to the manufacture's instructions. Basically the Pb^2+^ exposed cell lines (160 *μ*M) and control (HeLa cells) were seeding in a layer of 0.35% agar DMEM/FBS over a layer of 0.5% agar/DMEM/FBS. Cultures were maintained in a humidified 37°C incubator. On day 7 and day 30 after seeding, cells were fixed with pure ethanol containing 0.05% crystal violet and colony forming efficiency quantified by phase contrast microscope [28].

### 2.12. Statistical Analysis

The descriptive statistical analyses were performed using the software SPSS for Windows (Version 18.0, SPSS Predictive Analytics, Chicago). Data is presented as mean ± standard deviation (SD). Statistical comparisons were made using Student's *t*-test and *P* values *P* < 0.05 which were considered to be significant.

## 3. Results 

### 3.1. Morphology of Cells Exposed to Pb^2+^

**Figure 1:**
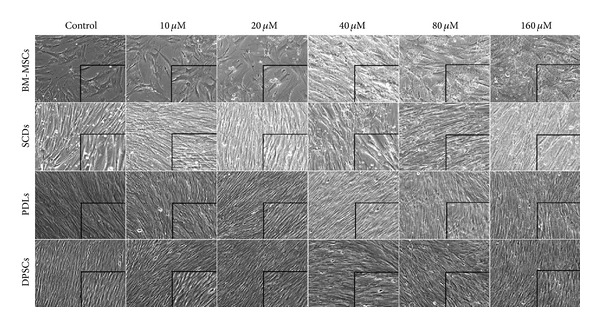
Cytotoxic effect of Pb^2+^ to bone marrow mesenchymal stem cells (BM-MSCs), deciduous stem cells (SCDs), periodontal ligament stem cells (PDLs), and permanent stem cells (DPSCs); phase contrast microscope; 10x magnification of BM-MSCs, SCDs, PDLs, and DPSCs in presence of various concentrations of Pb^2+^. In all experiments, the results represent average of five culture replicates with standard deviation and a representative photomicrograph was given for each experiment.

BM-MSCs, SCDs, DPSCs, and PDLs cultured in controlled conditions maintained a small and spindle-shape morphology and a similar observation was seen in cells exposed to 10 *μ*M of Pb^2+^ (Figure 1). However, BM-MSCs began to canalize and loose its cell texture and shape as well as displaying granule-like structures as the concentration of Pb^2+^ increased to 160 *μ*M. SCDs, DPSCs, and PDLs exhibited similar appearances but with a lesser degree.

### 3.2. Pb^2+^ Reduces the Proliferation Rate of BM-MSCs as Compared to Other Cell Lines

**Figure 2:**
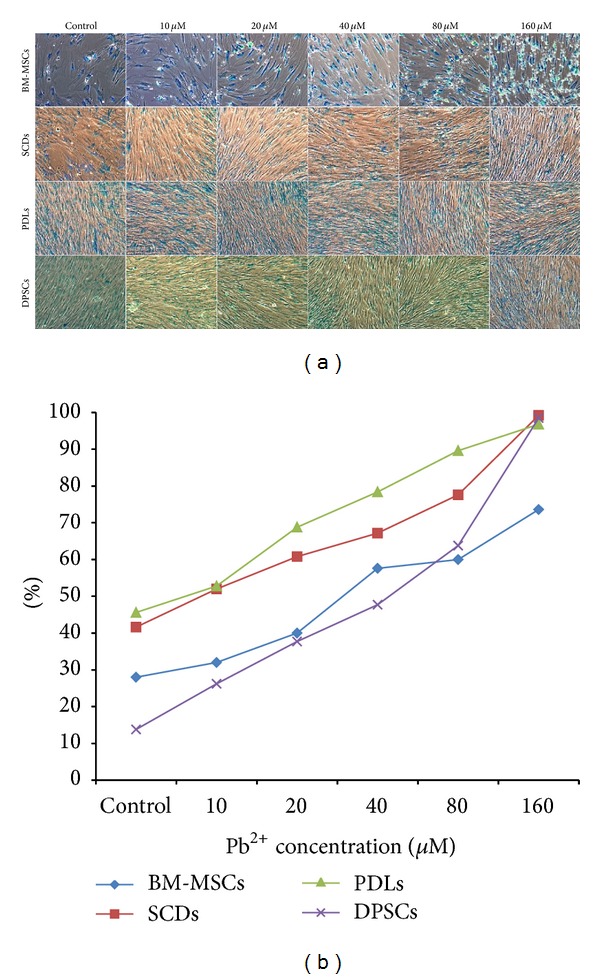
(a) Conventional qualitative SA-*β*-gal assay by X-gal staining of bone marrow mesenchymal stem cells (BM-MSCs), deciduous (SCDs), permanent (DPSCs), and periodontal ligament (PDLs) upon exposure to various concentrations of Pb^2+^. A locally region of senescence cell is shown; (b) quantification of percentage (%) of SA-*β*-gal positive cells exposed to various concentrations of Pb^2+^. In all experiments, the results represent average of five culture replicates with standard deviation and a representative photomicrograph was given for each experiment.

The 10 *μ*M Pb^2+^ treatment did not significantly (*P* > 0.05) inhibit the proliferation rate of any of the cell lines. However, a drastic inhibition of cell growth was observed in BM-MSCs exposed to 20 *μ*M Pb^2+^ (cell count: 1.3 × 10^6^ cells; MTT: 0.98 absorbance; LDH: 136%; *P* < 0.05) and up to 160 *μ*M Pb^2+^ treatment (cell count: 0.6 × 10^6^ cells; MTT: 0.24 absorbance; LDH: 164%; *P* < 0.05). While SCDs and DPSCs have constant rates of inhibition of cell growth, PDLs seem to be resistant to Pb^2+^ exposure. A small variation was observed in the control versus cells exposed to 160 *μ*M (cell count: 7.10 × 10^5^ cells; MTT: 0.78 absorbance; LDH: 146%; *P* > 0.05) (Table 1). This result was reflected in the aging of cells. The percentage of SA-*β*gal activity was increased in a concentration-dependent manner in all cell lines with the highest in BM-MSCs (ratio) followed by SCDs, DPSCs, and PDLs (Figure 2).

### 3.3. Pb^2+^ Alters the Expression of CD166 Marker Although Other Cell Surface Markers Remain Unchanged

Table 2 summarizes the immunophenotyping analysis of eight cell surface markers, namely, CD166, CD90, CD73, CD45, CD44, CD34, HLA-DR, and 7-AAD, from all sources of MSCs with the expression pattern being consistent throughout the Pb^2+^ concentrations. All four sources of MSCs in the control and Pb^2+^ treatments were found to be negative for the hematopoietic markers (CD34, CD45, and HLA-DR). On the other hand, a similar result was reported for the positive for cell surface markers (CD44, CD73, and CD90) among all the MSCs sources. Astonishingly, while the expressions of CD166 remain unchanged in BM-MSCs, DPSCs, and PDLs, it was lower in SCDs in Pb^2+^ treatments as compared to the control (see Supplementary Figures  1–4).

### 3.4. Multilineage Capability of All MSCs Cell Lines after Exposure to Pb^2+^

**Figure 3:**
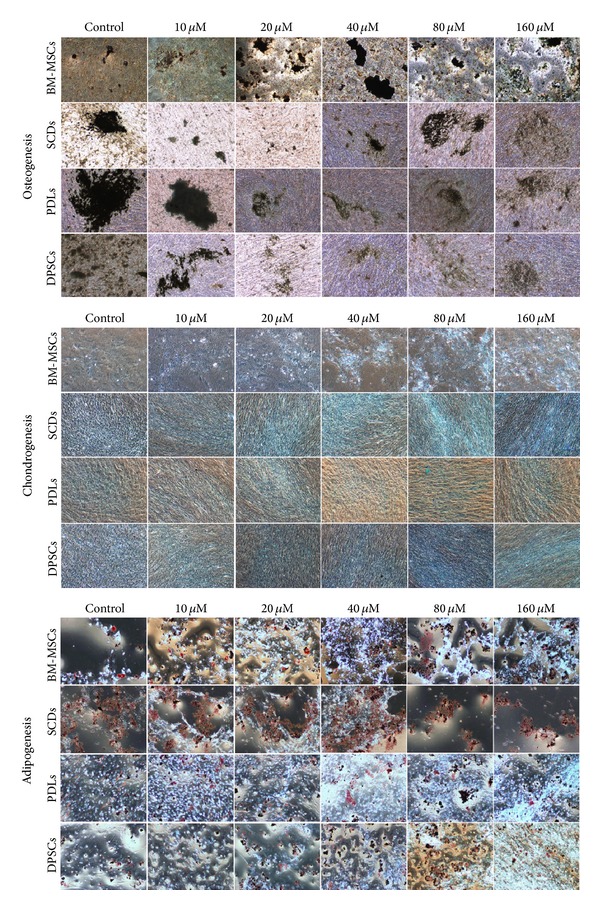
*In vitro* differential potentiality of bone marrow stem cells (BM-MSCs), deciduous stem cells (SCDs), periodontal ligament stem cells (PDLs), and permanent stem cells (DPSCs) in presence of various concentrations of Pb^2+^. Osteogenesis was confirmed by mineralized matrix deposition stained with von Kossa staining at day 21. Adipogenesis was detected by neutral oil droplet formation stained with Oil Red O at day 21. Chondogenesis was detected by the presence of proteoglycans stained with alcian blue dye at day 21. The results represent average of 3 independent culture replicates. A representative photomicrograph was given for each experiment.

We investigated the potential of BM-MSCs, SCDs, DPSCs, and PDLs to differentiate into osteogenic, chondrogenic, and adipogenic lineages at various concentrations of Pb^2+^ (Figure 3). Osteogenic differentiation was confirmed by the detection of the silver stained mineralized matrix in the control samples. While SCDs, DPSCs, and PDLs showed a weak deposition of calcium in the mineralized matrix only at the pretreatment with 160 *μ*M of Pb^2+^, BM-MSCs preexposed to Pb^2+^ concentration of 40 *μ*M have started to show a similar observation (Figure 3). Chondrogenic differentiation was detected by the presence of proteoglycans stained with alcian blue at day 21 in BM-MSCs, SCDs, PDLs, and DPSCs.

No significant differences were noticed between the control and pretreatment with Pb^2+^ in all MSC sources. Adipogenic differentiation was confirmed in BM-MSCs, SCDs, DPSCs, and PDLs by the accumulation of neutral lipid vacuoles indicated by the Oil Red O stain as shown in control (Figure 3). We discovered that for dental derived stem cells, the lipid vacuoles were observed until the highest concentration, although for BM-MSCs the neutral lipid droplet showed less accumulation of lipid vacuoles of cells exposed to 80 up to 160 *μ*M.

### 3.5. Gene Expression Profile of Stem Cells upon Exposure to Pb^2+^

**Figure 4:**
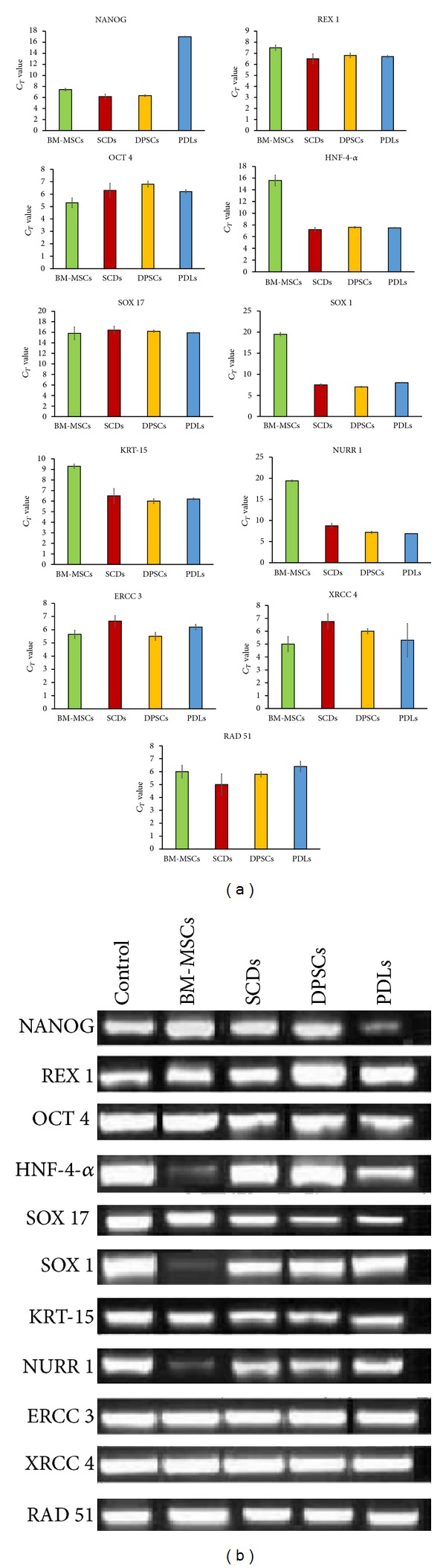
((a) and (b)) Gene expression analysis of bone marrow stem cells (BM-MSCs), deciduous stem cells (SCDs), periodontal ligament stem cells (PDLs), and permanent stem cells (DPSCs) for control and after treatment of Pb^2+^ at 160 *μ*mol. BM-MSCs, SCDs, DPSCs, and PDLs were tested against gene repair enzyme (ERCC3, XRCC14, RAD 51), stemness and self-renewal (Nanog, Rex 1, Oct 4), endoderm lineage (HNF-4*α*, SOX 17), and ectoderm lineage (SOX 1, KRT-15, NURR1). The lower a cycle threshold (*C*_*T*_) value is, the more copies are present in the specific sample. Values are presented after being normalized to 18s mRNA levels. The average of 3 replicates is displayed.

**Figure 5:**
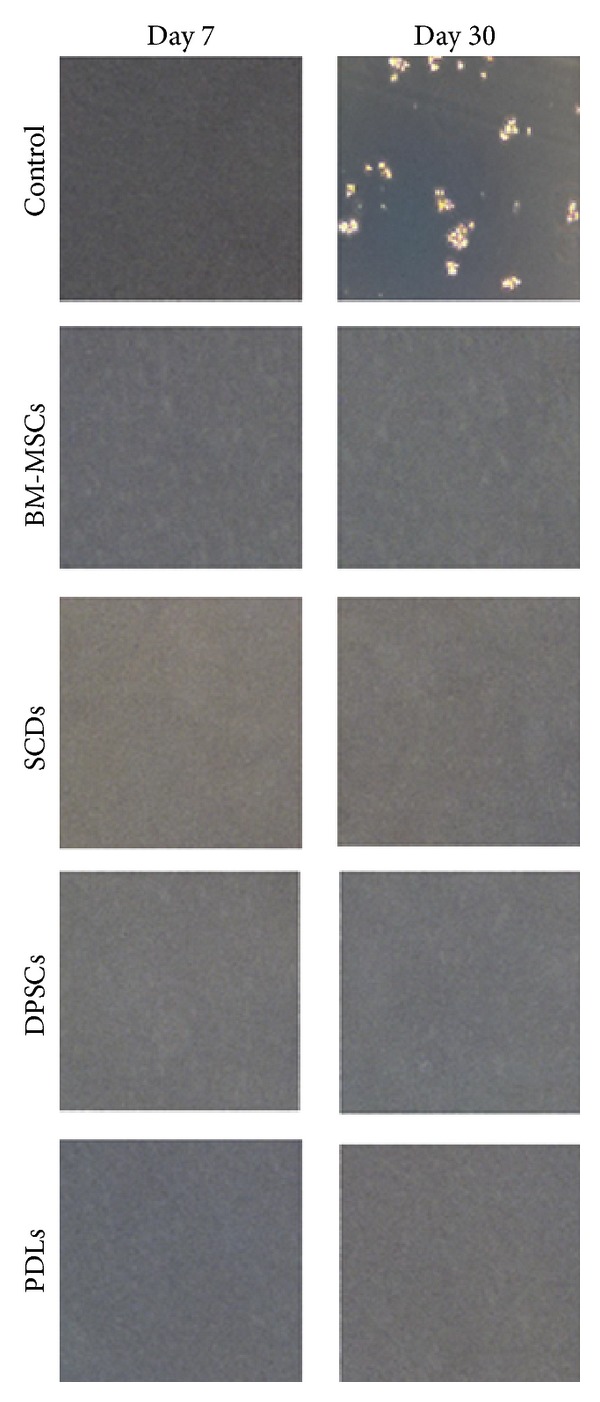
Soft agar assay of bone marrow stem cells (BM-MSCs), deciduous stem cells (SCDs), periodontal ligament stem cells (PDLs), and permanent stem cells (DPSCs) with HeLa cells as control after treatment of Pb^2+^ at 160 *μ*M. Phase contrast microscope; 10x magnification of BM-MSCs, SCDs, PDLs, and DPSCs in presence of various concentrations of Pb^2+^.

We further analysed the effect of Pb^2+^ on MSCs through gene expression analysis. The gene analysis was only carried out with cell lines treated with 160 *μ*M Pb^2+^ after taking into consideration that other concentrations of Pb^2+^ did not significantly impact the proliferation and differentiation of MSCs. Firstly, the effect of Pb^2+^ on the stemness and self-renewal (markers such as Nanog, Rex 1and Oct 4) were evaluated. Rex 1 and Oct 4 were uniformly maintained among the control and treated samples for all four sources of MSCs except for Nanog which was downregulated in PDLs compared to the other cell lines. Next, the effect of Pb^2+^ on MSCs in terms of lineage specificities was assessed. We observed a sharp decrease in expression of HNF-4*α* and SOX 17, early endoderm genes in the cells treated with Pb^2+^ from BM-MSCs. Nevertheless, the expression of HNF-4*α* and SOX 17 were similar in control and Pb^2+^ treated SCDs, DPSCs, and PDLs. In Pb^2+^ treated samples, the expression of SOX 1 and NURR1, in early ectoderm markers, was considerably downregulated in BM-MSCs, while the expression of KRT-15 was relatively stable among all sources. We also looked for signs of transformation in cells treated with Pb^2+^. We found that the expressions ERCC3, XRCC14, and RAD 51 remain unchanged in control and Pb^2+^ treated samples (Figures 4 and 5).

## 4. Discussion

For centuries, Pb^2+^ poisoning had posed a serious environment threat for human health in all societies [29]. Children have been more vulnerable to Pb^2+^ exposure than adults for many potential reasons, including their exposure to Pb^2+^, favoured by the habit of eating unhealthy food coupled with the evidence that a child's intestine absorbs Pb^2+^ much faster than that of an adult [30]. Among the detrimental effects of Pb^2+^ are the disruption of the peripheral and central nervous system [31, 32], blood, and skeletal systems [33, 34]. Pb^2+^ is stored mainly in the skeletal systems and reenters the blood circulation depending on bone turnover rates, which in turn depend on the type of bone (compact or trabecular) as well as on physiological and pathological conditions that affect the bone turnover rates [35, 36]. Given the multifaceted effects of Pb^2+^, it is important that we have a strong and continuous dataset for a better understanding of Pb^2+^ toxicity. Conventionally, laboratory animal-based systems have been used for toxicology studies, but conclusions based on animal testing raise questions due to numerous species-specific differences [37]. Alternatives such as whole embryo cultures and cellular models using primary or immortal cell lines have been developed. In this regard, stem cells from a dental origin could be an ideal cell source for Pb^2+^ screening and testing. Apart from noninvasive procedures in obtaining the cells, dental enamel is known to accumulate high amounts of Pb^2+^ on its surface [13, 38–43]. This artificial localization of Pb^2+^ turns dental enamel into a potentially interesting marker of exposure to Pb^2+^. Indeed, some studies have revealed that there is a relationship between the Pb^2+^ in surface enamel (Pb-enamel) and environmental lead exposure in permanent teeth [38–42] and in primary teeth [13, 43].

In this investigation, we have shown that Pb^2+^exposure inhibited adhesion with the highest being in BM-MSCs followed by SCDs, DPSCs, and PDLs as well as it increased the biological aging of the cells in a dose-dependent manner. Surprisingly, we noticed that dental derived stem cells are resistant to Pb^2+^ compared to BM-MSCs. One of the reasons is due to dissimilarities in age and gender of the donors. It should be noted that proliferation of stem cells gradually decreases as the age increases [44, 45]. But, at the mechanistic level, Pb^2+^ and ionic calcium (Ca^2+^) have a similar mechanism in entering and leaving the bone. Further, Pb^2+^ also follows the movement of Ca^2+^ in the body as it utilises the same ion transporter as calcium, acting like a competitive inhibitor [46]. The ion transporter and binding site of Ca^2+^ will recognize Pb^2+^ instead of Ca^2+^ and Pb^2+^ is released from the bone cells, along with Ca^2+^, when the bone is demineralised (as reviewed by [47]). But we postulate that the demineralisation process is slow in teeth allowing the cells of dental origin to adapt for more resistance to Pb^2+^ toxicity. In this study we identified that the concentration of Pb^2+^ (40 to 160 *μ*M) suppressed the proliferation of stem cells rather than inducing cell death. One possible explanation is that platelets are present in significant amounts in stem cells or bone marrow mononuclear cell cultures [48, 49] and Pb^2+^ is thought to influence platelets or lysate-based platelets by regulating the levels of growth chemotactic factors [50]. Thus, we speculate that Pb^2+^ inhibits growth factors related to the proliferation of stem cells resulting in slow growth of cells.

Next, we demonstrated that all four sources of MSCs were capable of differentiating into osteoblast, chrondocytes, and adipocytes in control samples. All four levels of Pb^2+^ exposed to MSCs cultured in an osteogenesis medium showed a reduction in osteogenesis differentiation capacity but a higher prevalence was observed in BM-MSCs.

Pb^2+^ has been shown in various tissues to block calcium signalling and inhibit Ca^2+^/phosphorylation and activation. There are also reports suggesting that Pb^2+^ suppressed the expression of osteogenic genes such as osteocalcin, alkaline phosphatase, and type I collagen [51].

In conclusion, we suggest that Pb^2+^ disturbed the osteogenic pathways, inhibiting the osteoblast differentiation. On the other hand, the results we obtained showed that the chondrogenesis was not greatly affected. [52, 53].

Pb^2+^ was found to induce chondogenesis in the presence of the transforming growth factor-*β* (TGF-*β*) and bone morphogenetic proteins (BMP) in MSCs [54–57].

The expression of CD166 was affected in Pb^2+^ exposed SCDs. This cell surface antigen is a known putative cancer stem cell marker derived in colorectal cancer [58]. It was speculated that the loss of expression of these markers contributed to metastasis. We suggest that Pb^2+^ did not significantly affect the rest of the cell surface antigen markers. This is because the target area for Pb^2+^ is in the chromatin and DNA of the cells which are located in the nucleus. Studies have been reported that Pb^2+^ interacts with histone protein in DNA and affects its integrity by forming cross links and eventually forms soluble complexes [59]. These complexes in turn may decrease the fidelity of DNA [60] and inhibits DNA and RNA synthesis, the process that introduces Pb^2+^ toxicity at the chromatin level [59]. Nevertheless, Pb^2+^ on cell surfaces is reported to increase B cell surface expression of murine MHC class II molecules [61].

Our results on gene expressions show that the gene repair enzyme did not significantly change the control and Pb^2+^ treated samples for all four MSC sources. This finding supports an earlier study stating that stem cells display an enhanced capacity to repair multiple forms of DNA damage including H_2_O_2_, UV-C, and ionizing radiation [62].

Recent studies have shown that a chronic low level of Pb^2+^ exposure may inhibit neurogenesis especially in the hippocampal formation and affect the differentiation/maturation of the newly generated neurons [63–65].

It has been reported that the heavy metals including Pb^2+^ interferes or inhibits gamma-aminobutyric acid (GABA), an inhibitory neurotransmitter binding to GABA receptors, a member of the ligand-gated ion channel super family [66]. Likewise, we hypothesize that activation of a similar pathway induced by GABA-mediated inhibition leads to selective silencing of neuronal markers such as SOX 1 and NURR1 in the present study. We observed a sharp decreased expression of HNF-4*α* in the cells treated with Pb^2+^ from BM-MSCs. In contrast, the expression of HNF-4*α*, a hepatic marker, had somewhat similar effects on SCDs, DPSCs, and PDLs. We observed that BM-MSCs have lower expression of HNF-4*α* marker compared to the other dental-derived MSCs sources. This is possibly due to the effect of Pb^2+^ displacing metal ions from proteins by altering the homeostasis of metals which could explain the effect of Pb^2+^ on gene expression. Our results are in agreement with the report demonstrating the regulation of Pb^2+^ in hepatocytes [67], indicating a hepatotoxic potential of Pb^2+^.

## 5. Conclusions 

We propose that the dental derived stem cells especially PDLs are an ideal source for *in vitro* heavy metal screening since it can withstand the toxicity of Pb^2+^ better than the other cell lines making it as one of the final frontiers to evaluate the extremisms of Pb^2+^ toxicity. Nevertheless, few factors need to be taken into consideration to avoid misinterpretation of data: (a) establishment of a good quality and quantity of cell lines is crucial to get a legitimate endpoint of heavy metal toxicity studies; (b) exposure of heavy metals should be prolonged to cover subchronic or chronic effects; (c) a combined battery of experiments covering physiology and biochemistry should be run concurrently to understand the synergic and agonistic effects of heavy metals.

## Supplementary Material

Supplementary Table 1 : The list of primers used in this study.
Supplementary Figure 1: Immunophenotype analysis of bone marrow mesenchymal stem cells (BM-MSCs).
Supplementary Figure 2: Immunophenotype analysis of permanent dental pulp stem cells (DPSCs).
Supplementary Figure 3: Immunophenotype analysis of deciduous dental stem cells (SCDs).
Supplementary Figure 4: Immunophenotype analysis of periodontal ligament stem cells (PDLs).

## Figures and Tables

**Table 1:** Changes in trypan blue dye exclusion assay, 3-(4,5-dimethylthiazol-2-yl)-2-,5-diphenyltetrazolium bromide (MTT) assay, and lactate dehydrogenase leakage (LDH) rate at various Pb^ 2+^concentrations (mean ± SD).

Index	Sample source	Control	10 µM	20 µM	40 µM	80 µM	160 µM
Trypan blue (in millions)	BM-MSCs	3.13 ± 0.07	2.85 ± 0.72	1.31 ± 0.33	1.21 ± 0.34	1.08 ± 0.69	0.65 ± 0.04
	SCDs	4.54 ± 0.66	4.25 ± 0.65	4.25 ± 0.42	4.21 ± 0.67	3.90 ± 0.60	3.45 ± 0.65
	PDLs	9.04 ± 0.11	8.88 ± 0.67	8.36 ± 0.91	8.31 ± 0.09	7.90 ± 0.55	7.08 ± 0.82
	DPSCs	5.33 ± 0.95	4.65 ± 0.53	4.44 ± 0.51	4.19 ± 0.91	4.07 ± 0.56	3.51 ± 0.67

MTT	BM-MSCs	1.07 ± 0.06	1.02 ± 0.02	0.98 ± 0.14	0.92 ± 0.05	0.82 ± 0.31	0.24 ± 0.03
	SCDs	1.14 ± 0.03	1.09 ± 0.15	0.97 ± 0.25	0.94 ± 0.02	0.89 ± 0.21	0.87 ± 0.04
	PDLs	1.24 ± 0.36	1.12 ± 0.66	1.10 ± 0.58	0.98 ± 0.29	0.80 ± 0.31	0.74 ± 1.45
	DPSCs	1.47 ± 0.74	1.27 ± 0.26	0.10 ± 0.23	0.80 ± 0.84	0.69 ± 0.08	0.55 ± 0.06

LDH (% of control)	BM-MSCs	100	120.45 ± 1.45	135.52 ± 1.58	146.48 ± 1.73	158.60 ± 1.46	164.42 ± 1.95
	SCDs	100	124.84 ± 1.86	134.15 ± 1.42	141.53 ± 1.36	149.59 ± 1.72	154.28 ± 1.31
	PDLs	100	116.35 ± 1.34	124.74 ± 2.04	136.84 ± 1.98	146.15 ± 2.14	158.26 ± 2.3
	DPSCs	100	124.52 ± 1.81	136.43 ± 1.47	142.55 ± 2.26	149.46 ± 2.41	153.98 ± 2.32

**Table:** (a)

BM-MSCs	7AAD	HLA-DR	CD34	CD45	CD44	CD73	CD90	CD166
Control	9.78%	20.22%	20.25%	21.81%	77.04%	74.99%	72.78%	51.50%
10 *μ*M	2.60%	17.32%	18.74%	18.34%	80.86%	74.48%	70.36%	46.44%
20 *μ*M	7.60%	25.24%	25.90%	23.34%	74.48%	67.78%	62.44%	54.72%
40 *μ*M	6.24%	17.94%	16.06%	18.66%	82.24%	77.70%	68.99%	57.22%
80 *μ*M	7.40%	20.89%	22.57%	21.54%	73.09%	74.27%	66.16%	67.62%
160 *μ*M	10.78%	24.89%	27.07%	26.32%	70.62%	68.45%	52.87%	68.19%

**Table:** (b)

DPSCs	7AAD	HLA-DR	CD34	CD45	CD44	CD73	CD90	CD166
Control	2.82%	24.21%	23.50%	23.94%	74.62%	76.49%	48.87%	53.03%
10 *μ*M	3.86%	20.11%	19.80%	20.16%	80.58%	80.72%	66.41%	69.32%
20 *μ*M	6.59%	30%	27.30%	28.49%	60.33%	74.84%	65.98%	71.66%
40 *μ*M	6.03%	30.64%	31.68%	31.32%	69.44%	66.48%	65.23%	67.20%
80 *μ*M	4.82%	33.42%	31.90%	32.63%	78.31%	69.22%	70.07%	66.02%
160 *μ*M	4.73%	31.04	32.05%	31.87%	66.45%	67.93%	60.14%	68.05%

**Table:** (c)

SCDs	7AAD	HLA-DR	CD34	CD45	CD44	CD73	CD90	CD166
Control	3.28%	19.82%	21.88%	21.71%	74.75%	75.40%	68.23%	47.84%
10 *μ*M	3.57%	21.88%	28.57%	27.57%	55.71%	69.13%	59.82%	53.25%
20 *μ*M	49.95%	30.18%	34.44%	30.51%	70.16%	72.23%	75.66%	41.45%
40 *μ*M	45.16%	43.36%	42.06%	42.06%	60.97%	65.94%	62.75%	41.83%
80 *μ*M	43.90%	50.52%	49.71%	49.60%	36.82%	48.71%	52.05%	30.39%
160 *μ*M	47.17%	44.86%	45.07%	45.20%	55.93%	63.16%	60.94%	41.94%

**Table:** (d)

PDLs	7AAD	HLA-DR	CD34	CD45	CD44	CD73	CD90	CD166
Control	7.74%	15.60%	15.57%	15.32%	78.33%	80.23%	77.15%	44.02%
10 *μ*M	2.66%	23.34%	23.23%	24.05%	72.54%	73.31%	71.62%	53.57%
20 *μ*M	3.48%	28.70%	28.24%	29.23%	66.42%	69.62%	62.05%	34.90%
40 *μ*M	3.01%	30.84%	31.68%	31.70%	62.60%	64.96%	58.66%	34.13%
80 *μ*M	5.79%	31.34%	33.75%	34.65%	60.92%	60.24%	58.84%	42.02%
160 *μ*M	7.65%	39.17%	38.43%	38.99%	55.80%	58.91%	70.07%	45.62%
