# Supplementary material for: Diverse Effects of Lead Nitrate on the Proliferation, Differentiation, and Gene Expression of Stem Cells Isolated from a Dental Origin
Source: ScientificWorldJournal. 2014 Jan 27;2014:235941. doi: 10.1155/2014/235941 (PMC3927845; doi:10.1155/2014/235941)
Supplement: Supplementary file 1 — Supplementary Table 1 : The list of primers used in this study. Supplementary Figure 1: Immunophenotype analysis of bone marrow mesenchymal stem cells (BM-MSCs). Supplementary Figure 2: Immunophenotype analysis of permanent dental pulp stem cells (DPSCs). Supplementary Figure 3: Immunophenotype analysis of deciduous dental stem cells (SCDs). Supplementary Figure 4: Immunophenotype analysis of periodontal ligament stem cells (PDLs). [file 235941.f1.pdf]

Table 1: List of primers used in this study

No	Gene	Forward and Reverse Primers (Forward and Reverse)	Annealing Temperature (◦C)	Base Pairs (bp)	Accession Number
1	RAD51	F: 5’-TTTGGAGAATTCCGAACT-3’<br>R: 5’-AGGAAGACAGGGAGAGTC-3’	58.5	588	NM_002875.3
2	XRCC4	F:5’-AAGATGTCTCATTCAGACTTG-3’<br>R:5’-CCGCTTATAAAGATCAGTCTC-3’	53.5	233	NM_022550.2
3	ERCC3	F:5’-CCAGGAAGCGGCACTATGAGG-3’<br>R:5’-GGTCGTCCTTCAGCGGCATTT-3’	63	171	NM_000122.1
4	NANOG	F:5’-TCCTCCATGGATCTGCTTATTCA-3’<br>R:5’-CAGGTCTTCACCTGTTTGTAGCTGAG3’	59	260	NM_024865.2
5	REX1	F:5’-GCGTACGCAAATTAAAGTCCAGA-3’<br>R:5’ –ATCCTAAACAGCTCGCAGAAT-3’	55	302	NM_174900.3
6	Oct-04	F:5’-CGACCATCTGCCGCTTTGAG-3’<br>R:5’- AAACCCTGGCACAAACTCCA -3’	61	619	NM_203289.3
7	HNF-4α	F:5’- GCTTGGTTCTCGTTGAGTGG-3’<br>R:5’-CAGGAGCTTATAGGGCTCAGAC-3’	58	762	NM_178850.1
8	SOX 17	F:5’-CGCACGGAATTTGAACAGTA-3’<br>R:5’-GGATCAGGGACCTGTCACAC-3’	58	146	NM_022454.3
9	SOX 1	F:5’- AAAACGAGGCGAGAGGCGAA -3’<br>R:5’-TGCCCTGGTCTTTGTCCTTCATCC-3’	62	896	NM_005986.2
11	KRT15	F:5’- CACAGTCTGCTGAGGTTGGA-3’<br>R:5’-GAGCTGCTCCATCTGTAGGG-3’	60	196	NM_002275.3
12	NURR1	F:5’-CGGACAGCAGTCCTCCATTAAGGT-3’<br>R:5’- CTGAAATCGGCAGTACTGACAGCG-3’	58	711	NM_006186.3
