# Supplementary figures and images for: Diverse Effects of Lead Nitrate on the Proliferation, Differentiation, and Gene Expression of Stem Cells Isolated from a Dental Origin
Source: ScientificWorldJournal. 2014 Jan 27;2014:235941. doi: 10.1155/2014/235941 (PMC3927845; doi:10.1155/2014/235941)

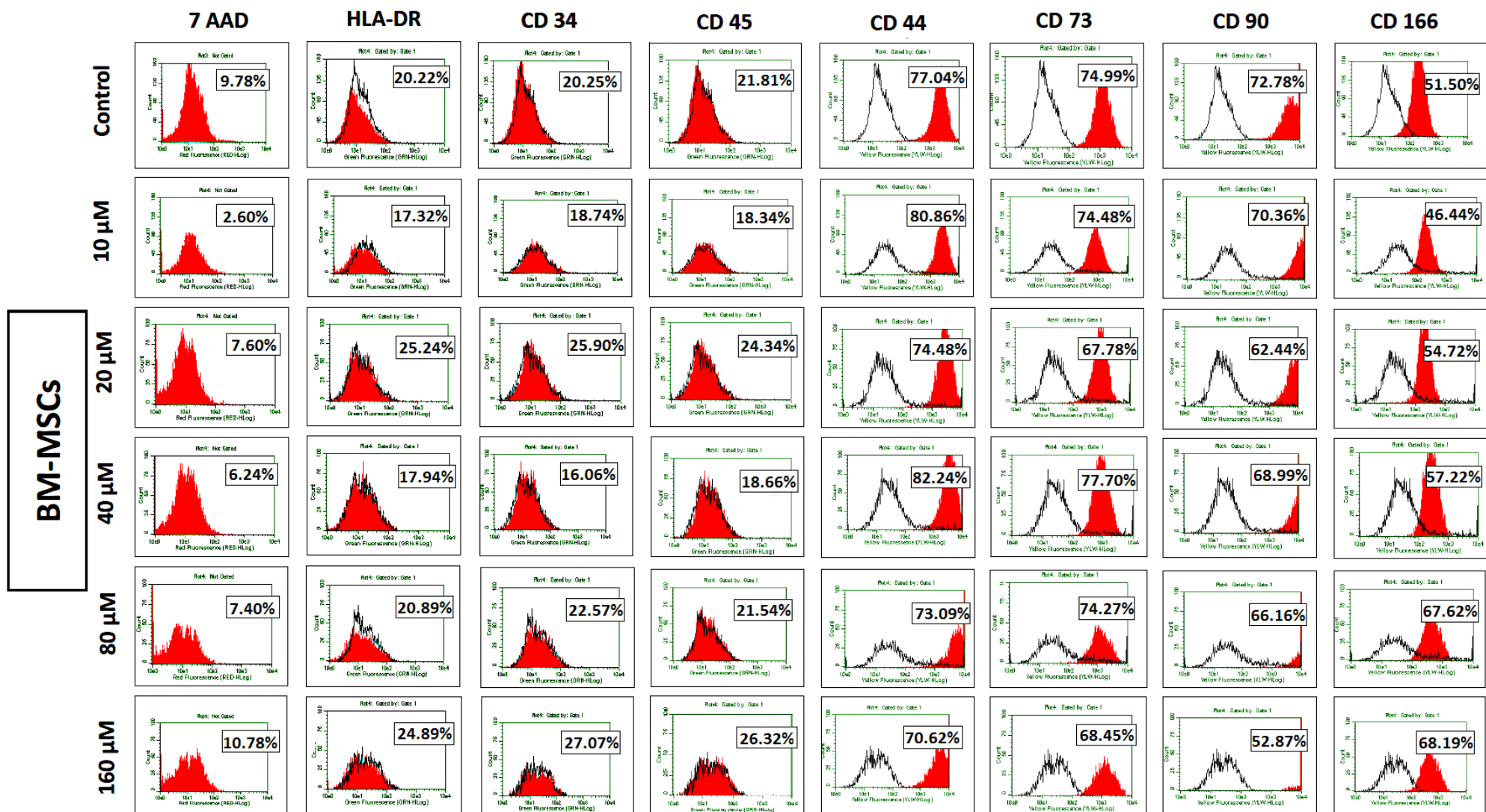

Supplement: Supplementary file 2 [file 235941.f2.pdf]

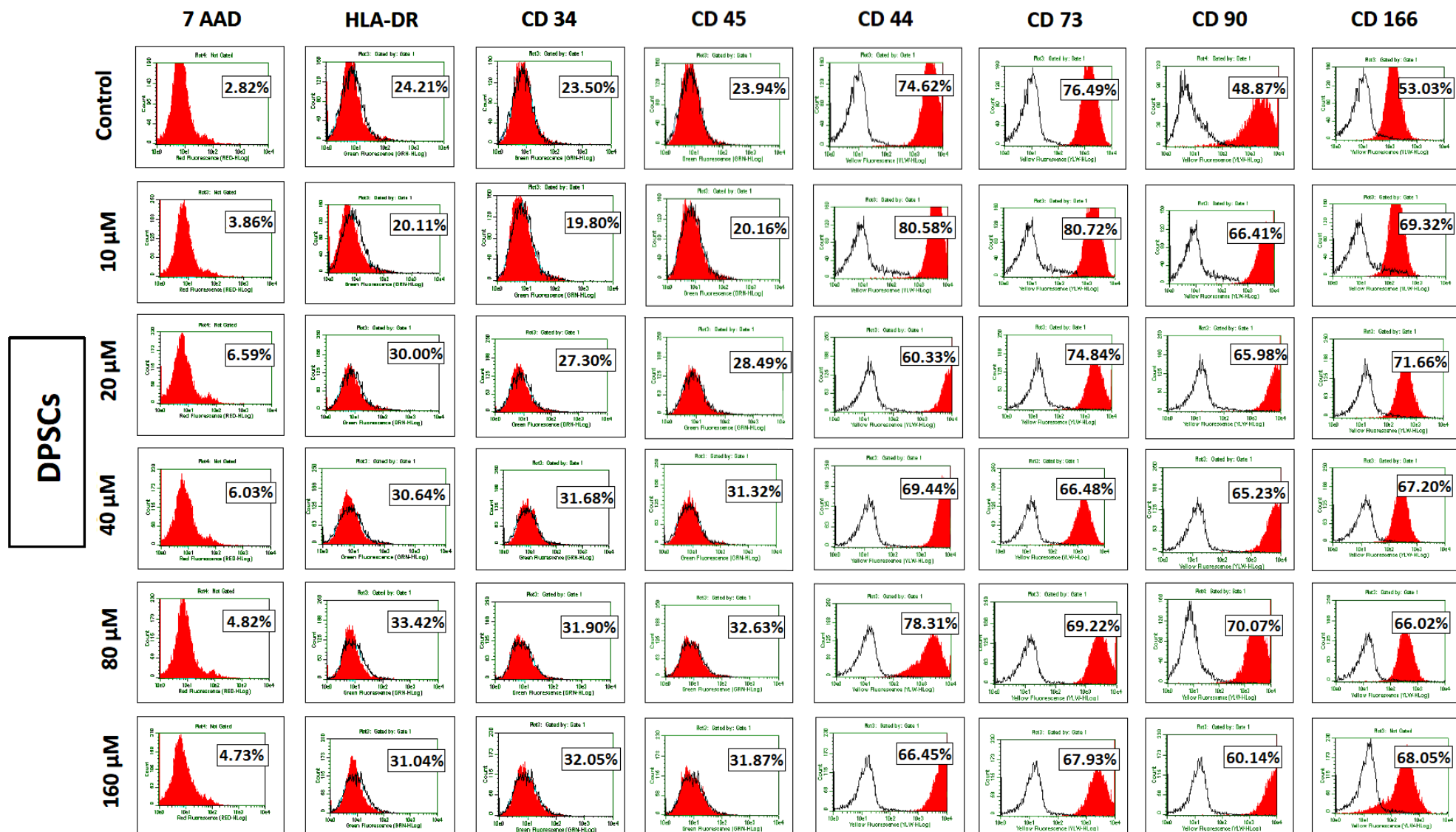

Supplement: Supplementary file 3 [file 235941.f3.pdf]

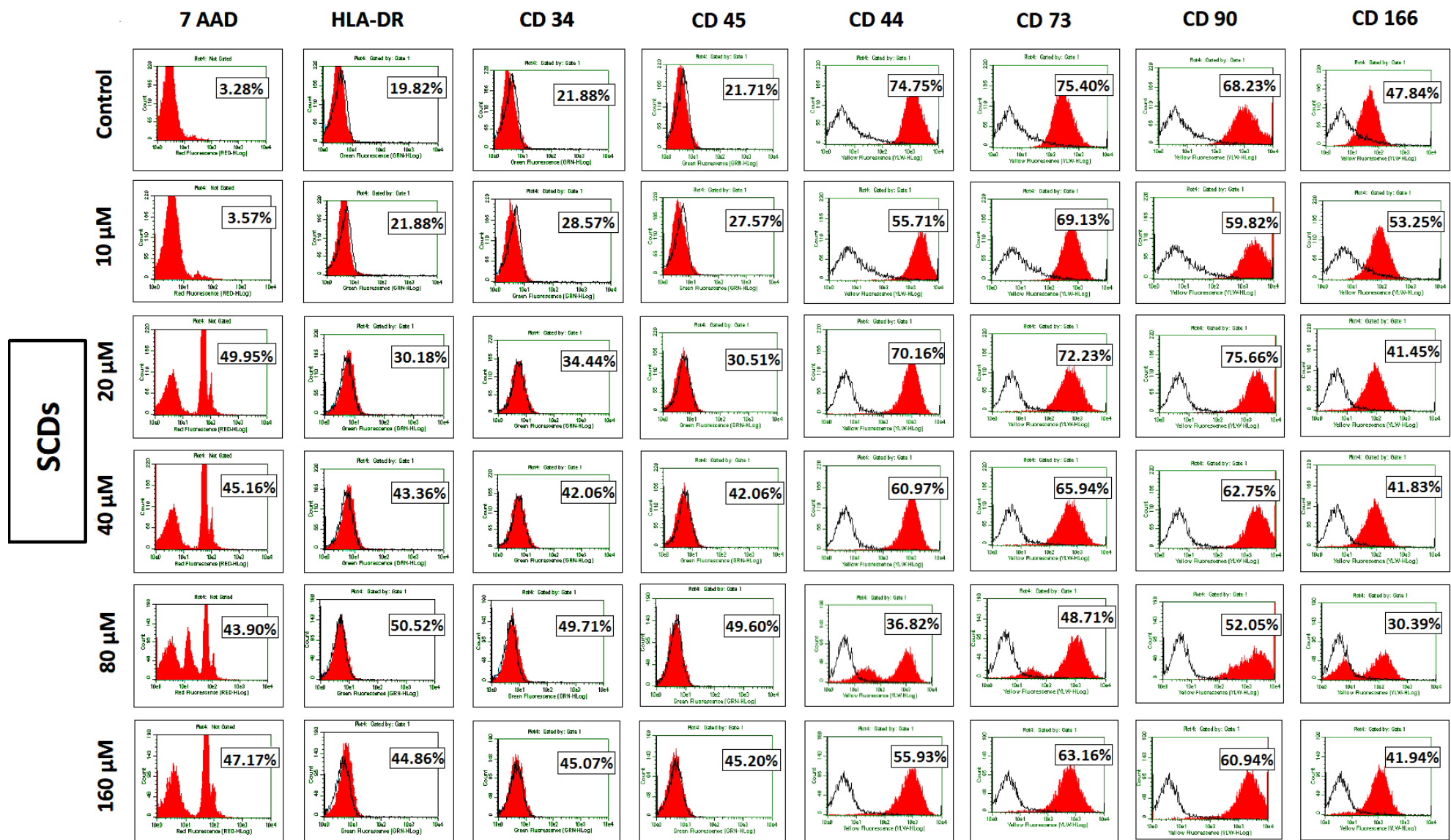

Supplement: Supplementary file 4 [file 235941.f4.pdf]

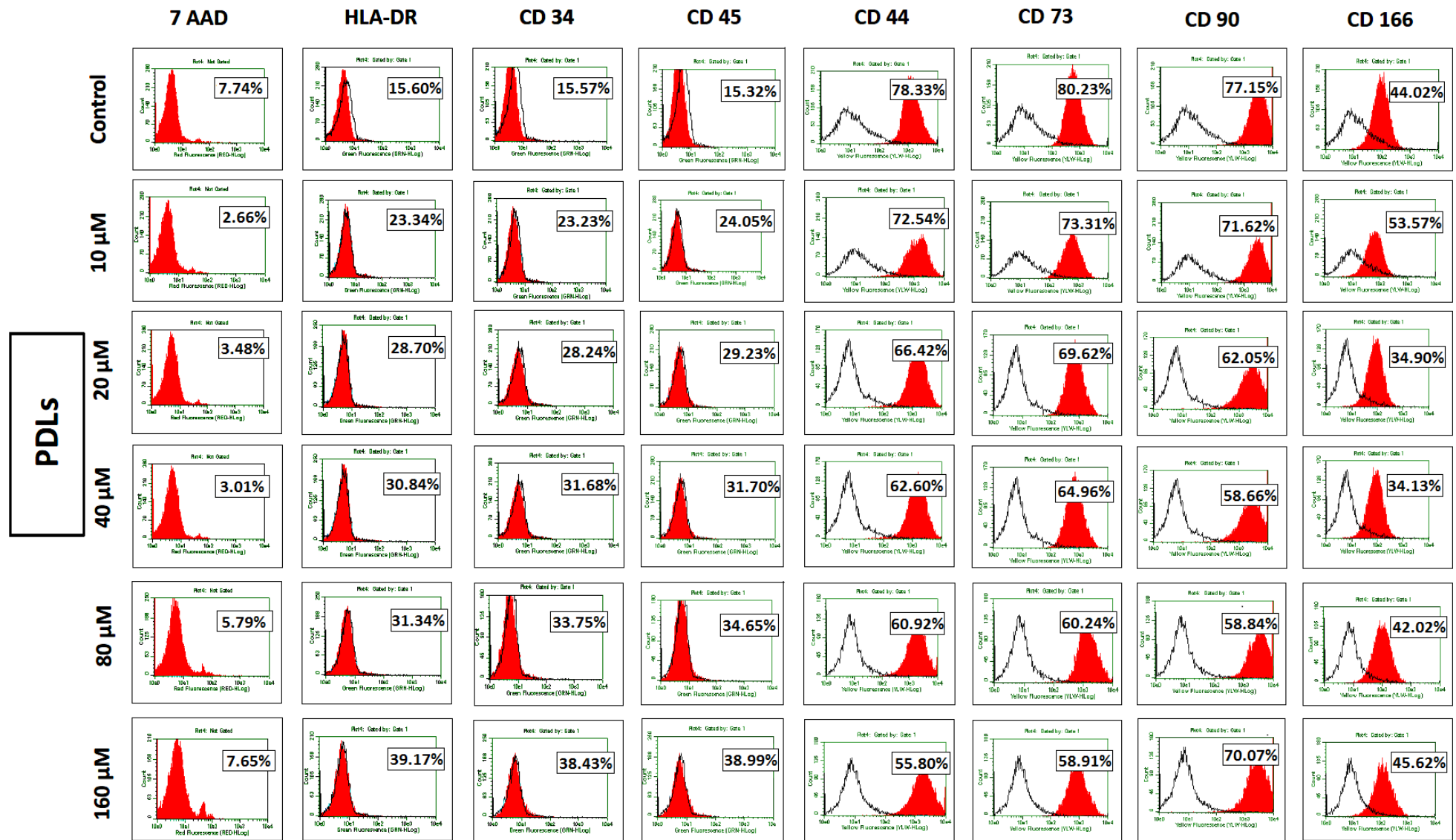

Supplement: Supplementary file 5 [file 235941.f5.pdf]
